# BuShenYiQi Granule Inhibits Atopic Dermatitis via Improving Central and Skin Hypothalamic -Pituitary -Adrenal Axis Function

**DOI:** 10.1371/journal.pone.0116427

**Published:** 2015-02-06

**Authors:** Lingwen Kong, Jingfeng Wu, Yanhua Lin, Genfa Wang, Jia Wang, Jiaqi Liu, Meixia Chen, Xin Du, Jing Sun, Jinpei Lin, Jingcheng Dong

**Affiliations:** 1 Department of integrated traditional Chinese and western medicine, Huashan Hospital, Fudan University, Shanghai, PR China; 2 Institute of integrated traditional Chinese and western medicine of Fudan University, Shanghai, PR China; 3 Department of Dermatology, Huashan Hospital, Fudan University, Shanghai, PR China; Medical University of Gdańsk, POLAND

## Abstract

**Background:**

Dysfunction of central and skin Hypothalamic-Pituitary-Adrenal (HPA) axis play important roles in pathogenesis of atopic dermatitis (AD). Our previous studies showed that several Chinese herbs could improve HPA axis function. In this study, we evaluated the anti-inflammatory effects of BuShenYiQi granule (BSYQ), a Chinese herbs formula, in AD mice and explored the effective mechanism from regulation of HPA axis.

**Methods:**

The ovalbumin (OVA) induced AD mice model were established and treated with BSYQ. We evaluated dermatitis score and histology analysis of dorsal skin lesions, meanwhile, serum corticosterone (CORT), adrenocorticotropic hormone (ACTH), corticotropin-releasing hormone (CRH) and inflammatory cytokines were determined by ELISA. The changes of CRH/proopiomelanocortin(POMC) axis elements, corresponding functional receptors and crucial genes of glucocorticosteroidogenesis in the skin were measured by quantitative real-time PCR and western blot, respectively.

**Results:**

The symptoms and pathological changes in skin of AD mice were significantly improved and several markers of inflammation and allergy descended obviously after BSYQ treatment. We found that AD mice had insufficient central HPA tone, but these conditions were markedly improved after BSYQ treatment. The AD mice also showed a disturbed expression of skin HPA. In lesion skin of AD mice, the mRNA and protein expressions of CRH decreased significantly, on the contrary, POMC and cytochrome P450 side-chain cleavage enzyme (CYP11A1) increased markedly, meanwhile, NR3C1 (mouse GR), CRHR2 and 11-hydroxylase type 1(CYP11B1) were reduced locally. Most of these tested indexes were improved after BSYQ treatment.

**Conclusions:**

AD mice displayed the differential expression pattern of central and skin HPA axis and BSYQ treatment significantly alleviated the symptoms of AD mice and presented anti-inflammatory and anti-allergic effects via regulating the expression of central and skin HPA axis.

## Introduction

Atopic dermatitis (AD) is a chronic inflammatory skin disease that causes significant impairment in quality of life. AD patients present obvious dysfunction of the Hypothalamic-Pituitary-Adrenal (HPA) axis by the reduced expression of serum glucocorticoid compared with normal persons, especially under the situation of stress[1,2]. The HPA axis is one of the most important parts in the nerve-endocrine-immune network which responds to various systemic stressors, such as psychological, physical injury and inflammatory factor[3]. Systemic and local infections activate the HPA axis and then POMC, POMC-derived peptides (ACTH, α-melanocyte-stimulating hormone and β-endorphin) and cortisol or corticosterone are released. One of the main pathogenetic mechanisms of allergic diseases is due to the low levels of cortisol or corticosterone and enhanced Th2 response[4,5,6,7].

The skin as an organ that was most frequently assaulted by a wide range of stressful environmental stimuli has developed a local defense system: peripheral HPA axis-like systems[8,9]. It has been reported that all regulatory elements of the central HPA axis were expressed in mammalian skin, including proopiomelanocortin-derived peptides[10], CRH and related peptides as well as the corresponding functional receptors, MC2R(the classical adrenocortical ACTH receptor) and glucocorticoid receptor NR3C1[11,12,13]. Recent study has shown that human skin cells have the capabilities of starting the steroidogenic pathway *de novo* from cholesterol because the skin expresses crucial genes of glucocorticosteroidogenesis enzymes including cytochrome P450 side-chain cleavage enzyme(CYP11A1), 11β-hydroxylase(CYP17), 21-hydroxylase(CYP21A2), and 11-hydroxylase type 1(CYP11B1)[14]. CYP11A1 is an important mitochondrial enzyme that starts steroidogenesis and CYP11B1 is another key enzyme which controls the synthesis of corticosterone[15]. Thus skin can be defined as an independent steroidogenic organ and malfunction of these steroidogenic activities can lead to inflammatory disorders. The peripheral HPA axis elements play important roles in the maintaining of skin local homeostasis [10]. Inflammatory stimulation of AD may also activivate the skin HPA axis elements; the expression sequence of peptides includes urocortin/CRH→POMC→ACTH. However, unlike the central HPA axis, the elements of peripheral HPA axis have more close connections between each other and more complicated functions[16]. Peripheral CRH is an important proinflammatory cytokine required for induction of the inflammatory response *in vivo* because acute stress response leads to increased skin vascular permeability and inflammation, largely through mast cell activation by CRH[17].

The most potent anti-inflammatory treatments available for AD is glucocorticoid-based therapy, however, the prolonged use of glucocorticoids can cause various side effects, therefore, it is imperative to explore other therapeutics which could increase the production or sensitivity of endogenous corticosteroid. Previously, we found that Shen-reinforcing and Qi-supplementing herbs could improve the symptoms of asthma rats and displayed anti-inflammatory effects via enhancing the expression of HPA axis[18,19]. We also found that the main components of these herbs could attenuate LPS-induced inflammatory responses via inactivating NF-kB *in vivo* and *in vitro* [20], reverse social defeat-induced down-regulation of glucocorticoid receptor and promote adrenal gland weight gain, significantly restore plasma corticosterone and ACTH level [21,44]. Therefore, we hypothesized that Shen-reinforcing and Qi-supplementing herbs might alleviate the symptoms of AD via improving central and skin HPA axis function, in addition to the anti-inflammatory and immunomodulatory effects. BuShenYiQi granule (BSYQ) composed of two Shen-reinforcing herbs and one Qi-supplementing herbs, Herba Epimedii, Astragalus membranaceus and Rehmannia Root, in a ratio of 4:6:3(w/w/w), is often used to treat inflammatory diseases in Chinese Medicine. Despite its frequently clinical use and efficacy, the molecular mechanism of this therapy is not fully understood. In this study, we evaluated the anti-inflammatory effect of BSYQ in AD mice and explore the effective mechanism from regulation of HPA axis.

## Materials and Methods

### Animals and groups

60 Female BALB/c mice were purchased from Shanghai SLAC Co. (Shanghai, China) and randomly divided into: sham group(control), atopic dermatitis group(AD model)(ovalbumin-induced), atopic dermatitis plus BSYQ 6.5g/kg/day (AD+6.5g/kg), atopic dermatitis plus BSYQ 13g/kg/day(AD+13g/kg), atopic dermatitis plus BSYQ 26g/kg/day(AD+26g/kg) and atopic dermatitis plus dexamethasone 1mg/kg/day(AD+dexm). The dose of mice used was based on a conversion table of equivalent effective dose ratios from human to animal. Mice were acclimated for 1 week to the housing condition before the experiments began and then 2–3 mice per cage housed in a laminar air flow room with a relative humidity of 55 ± 5%. Each room was maintained at 22 ± 2°C on a light-dark cycle of 12h light and 12h dark throughout the experiment.

### Ethics statement

Animal care and manipulation was in agreement with institutional guidelines, which are in accordance with the Guide for the Care and Use of Laboratory Animals. This study was approved by the Institutional Animal Care and Use Committee of Fudan University (Permit Number: 2013-03-HSYY-DJC-01). All surgery was performed under sodium pentobarbital anesthesia, and all efforts were made to minimize suffering.

### Induction of atopic dermatitis in the mouse skin

The protocol was performed based on previous research[22]. Briefly, 5 weeks female BALB/c mice were anesthetized with pentobarbital sodium (40–50 mg/kg i.p.)(St. Louis, MO, USA), then the back hair were shaved with an electric razor. After tape stripped 6 times with Tegaderm™ (3M Health Care, St Paul, MN, USA), the skin were challenged with 1 × 1cm sterile patches containing 100 μg OVA (Grade V, Sigma Inc., St Louis, MO, USA) or placebo (100 μL of PBS) for 3 weeks, which was secured to the skin also with Tegaderm™(3M Health Care, St Paul, MN, USA). The total time exposures to the patch were four-weeks separated by three-week intervals. Clinical symptoms of each mouse were evaluated as described previously[23], in brief, skin status was photographed every weeks after hair were removed, and degree of severity of erythema, edema, excoriation, hemorrhage and dryness on the skin were scored as 0 (none), 1(mild), 2 (moderate) and 3 (severe), respectively. The final score was the sum of all symptoms score. Scoring was performed by two different people who did not know the groups. The final score was taken as an average after every group scores were accumulated.

### Standardization of BSYQ

Three herbs were used in this study, including Radix Astragali (batch number: 1204811), Herba Epimedii (batch number: 1111018) and Rehmanniae Radix (batch number: 1203813). 3 herbs were purchased from Anhui Bencao Chinese Medicine Herb Pieces Co.,Ltd(Bozhou, China) and identified by the Testing Center of Jiangyin Tianjiang Pharmaceutical Co., Ltd(Jiangyin, China). Their extracts were prepared as follows: dried Astragalus membranes were soaked in water for 2h (1:8, w/v) and extracted twice with H_2_O at 100°C for 1h. The two decoction liquid extract were mixed. The dregs of the decoction were removed after filtering. The filtered liquid was concentrated to a clear cream whose relative density was 1.11–1.15 (60°C). After spray drying, the spray powder was mixed homogeneously. Herb of Epimedium or Radix Rehmanniae was also extracted by the method described above. Then three Chinese herbal medicines extracts were mixed for 30min in a ratio of 4:6:3(w/w/w). Spray powder was mixed homogeneously and made into 18–40 mesh particles. The granules were stored at 4°C and dissolved in distilled water at the desired concentrations before use. The three herbs are presented in **Table 1**.

**Table 1:** The BSYQ granule elements.

**Plant species**	**Family**	**Plant part**	**pinyin**
Epimedium L	Berberidaceae	Leaf	Yinyanghuo
Astragalus L	Leguminosae	root	HuangQi
Rehmannia	Scrophulariaceae	root	Shengdihuang

### The components analysis of BSYQ granule

The qualitative and quantitative analyses of chemical constituents of BSYQ granule were performed by HPLC-Q/TOF-MS method. Agilent Technonlogies Masshunter Workstation Acquisition Software (Rev. B. 05. 01) (LC/TOF) was used for the data acquisition. In addition, Q-TOF Agilent Technonlogies Masshunter Workstation Quantitative Analysis Software (Version B. 05. 02) was used for the data analysis. Errors were calculated by analyzing 3 batches of BSYQ granules. All reference standards of BSYQ granule were purchased from Pusi Bio-technology (Chengdu, China). The purities of all the compounds were above 98% based on HPLC-UV. HPLC analysis was done using Agilent 1260 series (Waldbronn, Germany) coupled with ESI ion source and photodiode array detector. Chromatographic separation was performed on Agilent Poroshell 120-C18 column (2.7×100 mm, 2.7 μm) at 35°C. The mobile phase was a mixture of water containing 0.1% formic acid (A) and acetonitrile (B). A linear gradient elution was conducted as follows: 0–8 min at 8–26% B, 8–16 min at 26–35% B, 16–23 min at 35–90% B, 23–26 min at 90% B, and 26–30 min at 8% B. The flow rate was 0.35 ml/min, and the injection volume was 3 μL.

### Histological analysis

The mice were anesthetized by pentobarbital sodium (60–70 mg/kg i.p.) and decapitated before the skin samples were removed. The lesional skin samples were fixed in 10% formaldehyde for 24h and embedded in paraffin. Skin sections were stained with haematoxylin and eosin. Epidermal thickness and various inflammatory cells were analysed in haematoxylin and eosin-stained sections and viewed under a magnification of ×200. 5 fields from each sample were randomly selected to measured epidermis thickness. CD4+ T cells were counted randomly in 10 high-power fields at 400× magnification. The skin sections were stained with 0.25% solution toluidine blue to measure the mast cell counts. The number of mast cells blinded in 10 sites chosen at random was counted.

### ELISA analysis

ELISA kits for IL-4, IL-5, INF-γ, CRH, ACTH, CORT, IgE and OVA-IgE were obtained from Xitang Biological Pharmaceutical Co. (Shanghai, China). The ELISA was performed in accordance with the manufacturer’s instructions. After the last treatment, mice were anesthetized by pentobarbital sodium (60–70 mg/kg i.p.) and 800–1000μL blood samples were collected at 8:00–9:00 by removal of eyeball prior to sacrificing which were allowed to clot for 30 min at room temperature, then centrifuged for 10 minutes at 3000rpm. The obtained volume of serum was 300–500μL in each mouse. The serum was stored at −80°C until use. The lower limits of detection of IgE, OVA-IgE, IL-4, IL-5, IFN-γ, corticosterone, ACTH and CRH were 2ng/mL, 2ng/mL, 4pg/mL, 10pg/mL, 2pg/mL, 0.15ng/mL,0.2ng/mL and 30pg/mL respectively.

### Real-time quantitative RT-PCR

Quantification was performed with a two-step reaction process: reverse transcription (RT) and PCR. Each RT reaction consisted of 0.5 μg RNA, 2 μL of PrimerScript Buffer, 0.5 μL of oligo dT, 0.5 μl of random 6 mers and 0.5 μL of PrimerScript RT Enzyme Mix I (TaKaRa Bio, Japan), in a total volume of 10 μL. Reactions were performed in a PCR system 9700(Applied Biosystems, USA) for 15 min at 37°C, followed by heat inactivation of RT for 5 s at 85°C. The 10 μL RT reaction mix was then diluted × 10 in nuclease-free water and held at −20°C. Real-time PCR instrument with 10 μL PCR reaction mixture that included 1 μL of cDNA, 5 μL of 2 × SYBR Green I Master (Roche, Swiss), 0.2 μL of forward primer, 0.2 μL of reverse primer and 3.6 μL of nuclease-free water. Reactions were incubated in a 384-well optical plate (Roche, Swiss) at 95°C for 10 min, followed by 40 cycles of 95°C for 10 s, 60°C for 30 s. Each sample was run in triplicate for analysis. At the end of the PCR cycles, melting curve analysis was performed to validate the specific generation of the expected PCR product. The primer sequences were designed in the laboratory and synthesized by Generay Biotech(Generay, PRC), based on the mRNA sequences obtained from the NCBI database. Primers used for PCR amplification are listed in **Table 2**. The expression levels of mRNAs were normalized to GAPDH and were calculated using the 2^−ΔΔCt^ method[24].

**Table 2:** List of primer sequences.

**Gene**	**Forward primer**	**Reverse primer**
CRH	TCAGAGCCCAAGTACGTT	AGGGACTTCTCTCAGGAT
CRHR1	ACAACTACTTCCACGTAACC	AGACGAACATCCACTTGC
CRHR2	GACCACGGGAAGTGAGTTA	TCCCAGGCATACTCTGATTT
POMC	AGAGGTTAAGAGCAGTGACTA	TCTCTCCCGCTATCTTTCCAA
MC2R	CTGCCTTTCTTATTCACAGTCT	AGTCCTCACATTTGTATGCTAT
CYP11B1	GGATGCTGTGAAGAGCTAAG	GACAGAAACCATTATGAAACGC
CYP11A1	GCGACAATGGTTGGCTAA	GTCCACGATGTAAACTGACT
NR3C1	TTTGATCTGTCAGCACTCAAG	TAACACTGAAACCAGGCACATA
GAPDH	CTTTGGCATTGTGGAAGGGC	CAGGGATGATGTTCTGGGCA

**Abbreviations**: **CRH**, corticotrophin releasing hormone; **CYP11A1 or P450scc**, cytochrome P450 side-chain cleavage enzyme; **CYP11B1**, 11β-hydroxylase type 1; **CRHR**, corticotropin-releasing hormone receptor (1 and 2); **POMC**, proopiomelanocortin; **NR3C1**, glucocorticoid receptor; **MC2R**, ACTH receptor.

### Western blot analysis

Lesion skin were homogenized in buffer with protease inhibitor and incubated for 30min at 4°C. Tissue debris was removed by centrifugation (12 000 × *g*, 15 min), then boiled 5 min at 98°C with SDS-PAGE loading buffer, and stored at −80°C until used. The protein concentration was determined by BSA method. 30μg of proteins were electroblotted onto a PVDF membrane, following the separation on a 10% SDS-polyacrylamide gel electrophoresis. The immunoblot was incubated 1h with 5% milk at room temperature, and then incubated overnight at 4°C with 1:1000 dilution of anti-CRH antibody(Santa Cruz Biotech, CA, sc-21000), 1:1000 dilution of anti-CRHR1 antibody(Santa Cruz Biotech, CA, sc-12383), 1:1000 dilution of anti-CRHR2 antibody(Santa Cruz Biotech, CA, sc-20550), 1:1000 anti-CYP11A1 antibody(Santa Cruz Biotech, CA, sc-292456), 1:1000 dilution of anti-CYP11B1 antibody(Santa Cruz Biotech, CA, sc-32372) and 1:1000 dilution of β-actin antibody(Santa Cruz Biotechnology, sc-130656), respectively. Blots were washed three times with Tween-20/Tris-buffered saline (TTBS) and then incubated with a 1:10000 dilution of HRP-conjugated secondary antibody for 1h at room temperature. Blots were again washed three times with TTBS and then developed by enhanced chemiluminescence. Band intensities were quantified using ImageJ analysis software. The optical density for the target proteins were shown as a proportion of β-actin optical density.

### Statistical analysis

The data are expressed as the means±Standard Deviation (SD). SPSS 17 software(SPSS Inc., Chicago, USA) was used for statistical analysis. The significance of the differences was determined by one-way ANOVA, comparisons of parameters between 2 groups were performed with LSD or Games-Howell test. The data analysis of q-PCR gene expression levels of normal mice were set at 1-fold, and gene expression changes of other groups were expressed as the normalized “fold” change in mRNA expression compared with the normal mice. Appropriate analyses of variance were also performed to confirm significant differences between groups.

## Results

### The chemical constituents of BSYQ granules

**Figure 1:**
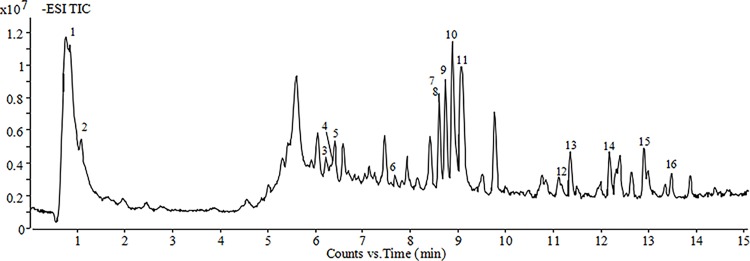
Total ion chromatography of BSYQ granule by HPLC-Q/TOFMS. (1) Catalpol 1.97±0.21μg/mg; (2) Leonuride 0.221±0.009μg/mg; (3) Calycosin-7-O-β-D-glucoside 0.183±0.003μg/mg;(4) Hyperoside 0.127±0.003 μg/mg; (5) Acteroside 0.246±0.005μg/mg; (6) Formononetin-7-O-β-Dglucoside,0.0946±0.002 μg/mg; (7) EpimedinA 0.472±0.010μg/mg; (8) Calycosin,0.167±0.011μg/mg; (9) Epimedin B, 1.59±0.02μg/mg; (10) Epimedin C, 3.01±0.22μg/mg; (11) Icariin 3.14±0.08μg/mg;(12)Formononetin 0.0943±0.0022μg/mg; (13) Astragaloside IV, 1.17±0.10μg/mg; (14) Astragaloside II, 0.447±0.003μg/mg; (15) Baohuoside-I, 0.964±0.033μg/mg; (16) Astragaloside I, 0.479±0.003μg/mg.

The qualitative and quantitative analysis of chemical constituents of BSYQ granules were performed by LC-ESI-Q-TOF-MS. There were71 bioactive components in BSYQ granule[25] and the 16 main active chemical constituents were shown in **Fig. 1**.

### BSYQ treatment alleviated AD-like symptoms

**Figure 2:**
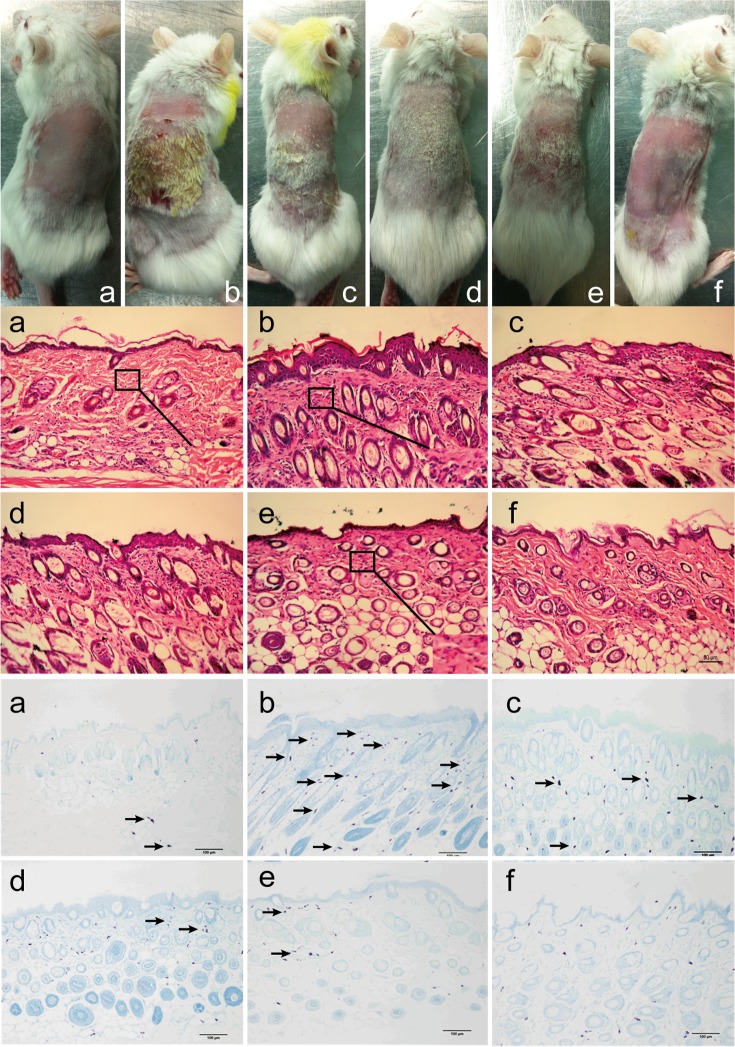
The inhibitory effects of BSYQ on OVA-induced AD skin symptoms and histological analysis of skins. **Above:** Example of the clinical severity of AD-like skin lesions. Skin status was photographed every week after hair was removed, and degree of severity of erythema, edema, excoriation, hemorrhage and dryness on the skin was scored as 0 (none), 1(mild), 2 (moderate) and 3 (severe). The final score was the sum of all symptoms score.

**Figure 3:**
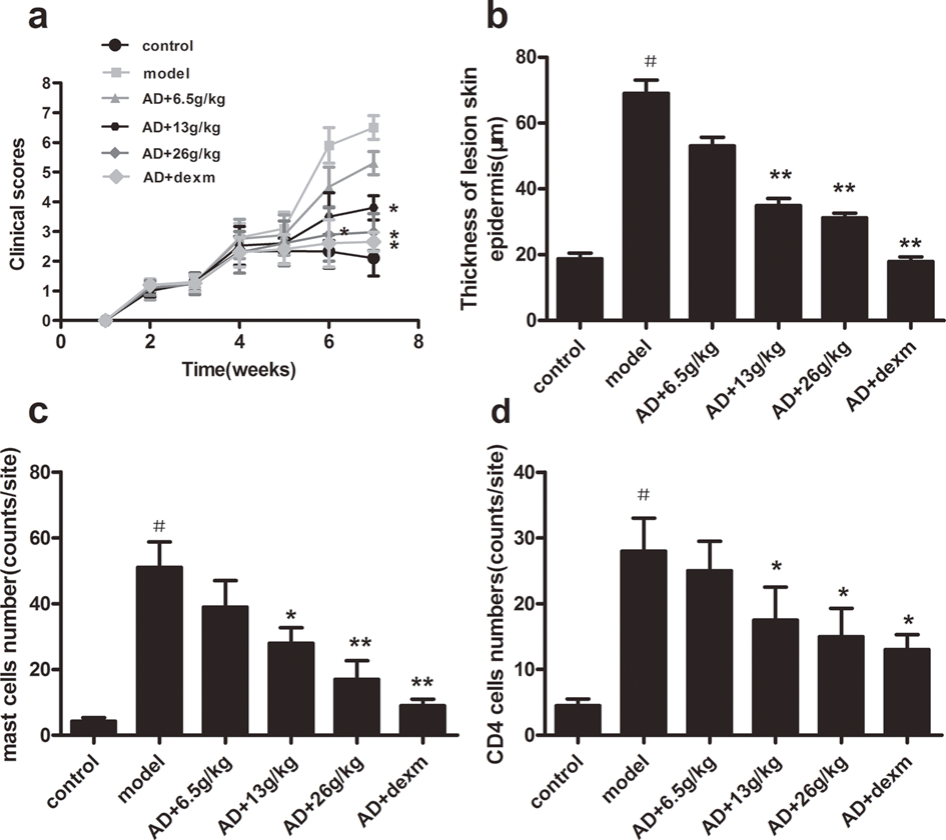
Clinical scores, epidermal thickness and the infiltration of immune cells in AD mice. **a:** Clinical scores; **b**: Thickness of lesion skin epidermis; **c**: Mast cells number; **d**: CD4 cell number. The dermatitis score were measured at 1,2,3,4,5,6,7 week after AD induction and defined as the sum of scores for five clinical criteria: erythema, edema, excoriation, hemorrhage and dryness; 5 fields from each sample were randomly selected to measured epidermis thickness; CD4+ T cells and mast cells were counted randomly in 10 high-power fields. The final score was taken as an average after every group scores were accumulated. Data are presented as mean ± SD (n = 6). ^#^ p<0.05 vs. control group, *p<0.05 vs. AD group, **p<0.01 vs. AD group.

To evaluate the effects of BSYQ against AD-like skin lesion, AD mice were treated with 6.5g/kg, 13g/kg or 26g/kg BSYQ by gavages daily during the period of the skin sensitization with OVA, meanwhile, AD mice were also treated with dexamethasone 1mg/kg per day as a positive control. BSYQ treatment significantly reduced OVA-induced AD symptom severity in a dose-dependent manner (**Fig. 2 above** and **Fig. 3A**).

**Middle and below:** Histological analysis of skin. Representative photomicrographs of skin sections were stained with hematoxylin and eosin (middle) or toluidine blue (below) (original magnification: HE×200; toluidine blue×100). There are marked hyperplasias of the epidermis and excessive infiltration of inflammatory cells into the dermis in AD group, BSYQ reduced the thickness of the epidermis and numbers of lymphocyte and mast cells. Further magnification of the thin black-bordered box in the inset region shows the presence of the lymphocyte cells (middle) and the arrows show the presence of mast cells (blow). Scale bar = 50μm (HE) and 100μm (toluidine blue). **a**: control; **b**: AD model; **c**: AD+6.5g/kg; **d**: AD +13g/kg; **e**:AD+26g/kg; **f**:AD+ dexamethasone.

### Effects of BSYQ treatment on the inflammation of AD

The thickness of the epidermis and dermis were significantly higher in mice with atopic dermatitis, as compared with that of normal mice and excessive infiltration of inflammatory cells into the dermis was observed in AD mice (**Fig. 2**). BSYQ treatment could significantly reduce the thickness of epidermis and the numbers of lymphocyte and mast cells in the dermis in a dose-independent manner. (**Fig. 3B, Fig. 3C** and **Fig. 3D**).

### Effects of BSYQ treatment on inflammatory markers

**Figure 4:**
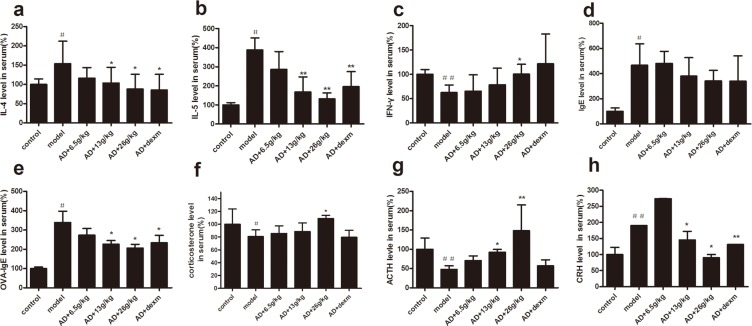
Serum IL-4 (a), IL-5(b), IFN-γ(c) IgE(d), OVA-IgE(e), Corticosterone(f), ACTH(g) and CRH(h) levels. Blood samples were collected at 8:00–9:00 by removal of eyeball prior to sacrificing which was allowed to clot for 30 min at room temperature, then centrifuged for 10 minutes at 3000rpm. The ELISA was performed in accordance with the manufacturer’s instructions. Data are presented as mean ± SD (n = 6). ^#^ p<0.05 vs. control, ^##^ p<0.01vs. control,*p<0.05 vs. AD group,**p<0.01 vs. AD group.

In order to evaluate the effects of BSYQ treatment on inflammatory responses in AD mice, we measured Th1 and Th2 cytokines in serum. As compared with blank control group, Th2 cytokines (IL-4, IL-5) increased (p<0.05 for both, **Fig. 4A** and **Fig. 4B**) and Th1 cytokine (IFN-γ) reduced significantly (p<0.01, **Fig. 4C**) in the AD group. The IL-4 and IL-5 levels in serum were significantly suppressed by BSYQ 13g/kg and 26g/kg treatment, on the contrary, IFN-γ levels were markedly increased in BSYQ 26g/kg treatment group, as compared with that of AD control mice (p<0.05).

In order to investigate the effect of BSYQ on allergic response of AD, the total IgE and OVA-IgE in serum were measured. Both of them were up-regulated in AD mice, as compared with that of normal mice (P<0.05 for both, **Fig. 4D** and **Fig. 4E**), and BSYQ (13g/kg and 26g/kg) treatment significantly reduced the serum levels of OVA-IgE in a dose-dependent manner, as compared with that of AD control mice (P<0.05 for both, **Fig. 4D** and **Fig. 4E**).

### Effects of BSYQ treatment on central HPA axis

We measured the level of serum CRH, ACTH and corticosterone to evaluate the central HPA axis function. As compared with blank control group, the concentrations of ACTH and corticosterone significantly reduced in the AD group, however, BSYQ (13g/kg, 26g/kg) treatment significantly increased the levels of ACTH (p<0.05 p<0.01) and corticosterone(p<0.05) in serum of AD mice **(Fig. 4F** and **Fig. 4G**). On the contrary, the CRH concentrations significantly increased in the AD mice (p<0.01), BSYQ 13g/kg and 26g/kg treatment decreased the levels of CRH in AD mice (p<0.05 and p<0.01 respectively, **Fig. 4H**).

### Effects of BSYQ treatment on skin HPA system

#### Gene expression levels in skin

**Figure 5:**
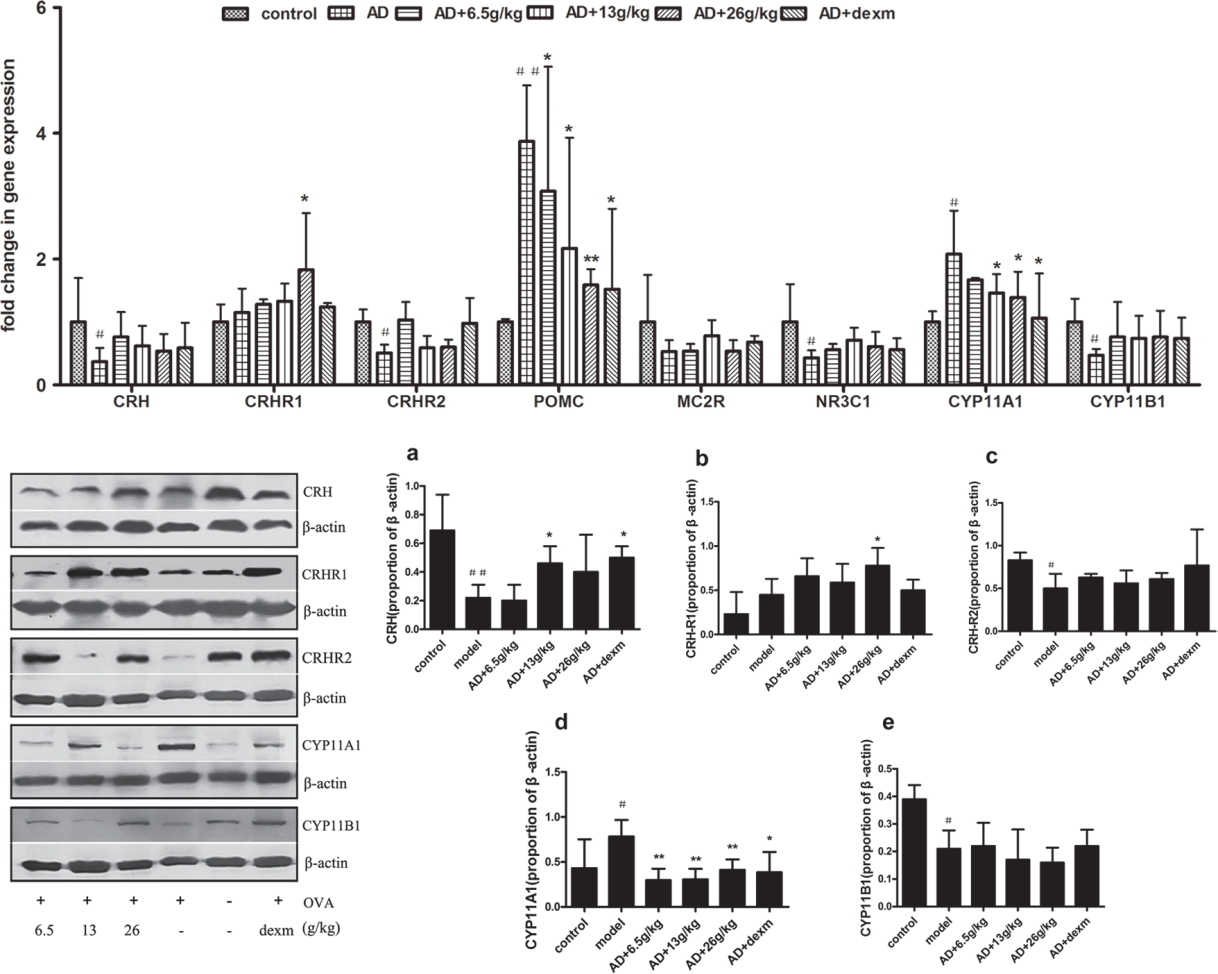
The mRNA and protein expression of skin HPA axis. **Above**: The mRNA levels of HPA hormones and their receptors in the skin in comparison to normal mice. Gene expression levels of normal mice were set at 1-fold, and gene expression changes in AD group, BSYQ groups and Dexm group were expressed as the normalized “fold” changes in mRNA compared with those of normal mice(n = 6). **Below:** The protein expression of CRH(a), CRHR1(b), CRHR2(c), CYP11A1(d) and CYP11B1(e). Quantitative measured by Western blotting, relative to β-actin. Statistical analysis for significant differences in expression between groups was performed on the data using ANOVA(n = 6). *p<0.05 vs. AD group; **p<0.01 vs. AD group; ^#^p<0.05 vs. control group; ^##^p<0.01 vs. control group. **Abbreviations: AD**, atopic dermatitis; **HPA axis**, hypothalamic-pituitary-adrenal axis; **CRH**, corticotrophin releasing hormone; CYP11A1 or P450scc, cytochrome P450 side-chain cleavage enzyme; **CYP11B1**, 11β-hydroxylase type 1; **CRHR**, corticotropin-releasing hormone receptor (1 and 2); **POMC**, proopiomelanocortin; **NR3C1**, glucocorticoid receptor; **MC2R**, ACTH receptor.

We detected the mRNA expression of HPA-related genes: CRH, POMC, CRHR1, CRHR2, NR3C1, MC2R, CYP11A1 and CYP11B1 by real-time PCR. The cut off criteria of the fold change > 1.5 or < 0.52 and P < 0.05. The expression of CRH mRNA in AD group were reduced to 0.37-fold, as compared with that of blank control (p<0.05) and the CRH mRNA expression have no changes after BSYQ treatment (Fig. 5 above). As compared with blank control mice, there were 3.87- fold increases of POMC expression levels in lesion skin of AD mice. After treatment with BSYQ, the expressions of POMC mRNA were significantly down-regulated in a dose-dependent manner (3.08-, 2.16- and 1.59-fold for 6.5g/kg, 13g/kg and 26g/kg, respectively, Fig. 5 above).

With the exception of CRHR1, the receptors of stress hormones were mainly decreased in AD mice, as compared with control mice. There was a minor increase in CRHR1 mRNA expression in AD lesions (1.15-fold), while CRHR2, Mc2r and NR3C1 expressions significantly down-regulated in AD mice (0.51-, 0.52- and 0.43- fold, respectively). BSYQ treatment dose-dependently increased CRHR1 mRNA expressions in the AD lesion skin and the BSYQ 26g/kg treatment group reached at the peak level (p<0.05).The expressions of CRHR2 and NR3C1 were down-regulated in AD mice (p<0.05 for both), while, BSYQ did not display obvious effects on both of them.

The key enzyme of steroidogenesis, CYP11A1, had a 2.08-fold expression in the AD mice, as compared with control group (P = 0.05). It was significantly reduced after BSYQ 13g/kg and 26g/kg treatment (p<0.01 for both). In contrast, we found that CYP11B1, another key enzyme of steroidogenesis, had a 0.47-fold reduction in the AD mice (p<0.05) (**Fig. 5 above**).

#### Protein expression levels in skin

To further evaluate the skin HPA function, the protein expressions of CRH, CRHR1, CRHR2, CYP11A1 and CYP11B1 were performed by western blot. As compared with blank control mice, the CRH protein significantly reduced in the lesions of AD mice (Fig. 5 below). Unlike the effect on CRH mRNA, the BSYQ (13g/kg and 26g/kg) treatment significantly increased the expression of CRH protein (P<0.05 for both, Fig. 5 below). On the contrary, AD skins showed over-expressed CYP11A1 proteins, while BSYQ (6g/kg, 13g/kg and 26g/kg) treatment markedly down-regulated the expression of CYP11A1 in the skin lesions (P<0.01 for three, Fig. 5 below). As compared with blank control group, the CYP11B1 and CRHR2 protein levels significantly decreased in AD mice (p<0.05 for both, Fig. 5 below). BSYQ treatment could not alter the levels of CYP11B1 and CRH-R2 protein, as compared with AD model group.

## Discussion

The abnormal HPA axis function is one of the most important pathogenesis of atopic dermatitis[26]. In allergic patients, the failure to produce a sufficient glucocorticoid concentration might result in an imbalance of Th1/Th2 and trigger allergic inflammation[27,28]. Other research has reported that in chronic inflammatory conditions, the HPA axis was firstly activated by pro-inflammatory cytokines but in the longer-term, it was exhausted and associated with reduced ACTH and glucocorticoid[29]. Supplementation of exogenous steroids is one of the most powerful therapeutics in AD treatment; however, the prolonged use of glucocorticoids can cause various systemic side effects[30], the aim of the study was to find an alternative treatment with fewer side effects.

In this study, we confirmed that AD mice exhibited down-regulated central HPA axis function, disturbed Th1/Th2 response, increased IgE and OVA-IgE concentration in serum. We demonstrated that BSYQ treatment could alleviate AD-like symptoms, improve central HPA axis function and increase endogeneous corticosteroid levels. BSYQ treatment could also inhibit Th2 function and stimulate Th1 response. The IgE and OVA-IgE levels were attenuated by BSYQ treatment. This kind of immunomodulatory effects of BSYQ were verified by another study. In that study, we found that BSYQ could down-regulate Th2-Th17 cell proportions and up-regulate Th1 cell proportion in asthma[31]. In addition, other researchers reported that Chinese herb formula, ASHMI, also had similar immunomodulatory effects in allergic diseases[32]. Mast cells can rapidly induce multiple tissue effects in skin that include vasodilation, angiogenesis, and proinflammatory activities[26]. Our study demonstrated that BSYQ markedly reduced the numbers of mast and CD4+ cells in the lesional skin. This was one of the important anti– inflammatory mechanisms of BSYQ.

Importantly, we have demonstrated the disturbed expression of skin HPA axis elements and steroid synthesis-regulators in the lesional skin of AD mice. In the central HPA axis, its communication of the crucial regulatory elements is unidirectional and linear, on the contrary, all of the elements of the skin HPA-like system are primarily found in the same organ, this generates multidirectional communications that are nonlinear, but with possible feedback and feedforward mechanisms operating at different levels of coordinating points, and in this complicated system, CRHR1 is at center position[16]. In addition, different mouse species display differences in HPA axis activity[33, 34], however, BALB/c mouse has more sensitive HPA response to stress[35], so we chose this stain as AD model. In this study, we found that the expression of skin HPA axis elements, POMC and CYP11A1, in AD mice were higher than that of control group, while the CRH, NR3C1 and CYP11B1 mRNA were significantly down-regulated and the receptors of ACTH, MC2R, were slightly down-regulated. However, CRHR1 did not show obvious changes. Malfunction of these elements can lead to autoimmune diseases and this is probably the most important cause of atopic dermatities[16]. The described differential expression of HPA axis elements induced in AD lesions offers distinct but overlapping mechanisms by which local neuroendocrine pathways maintain the protection of cutaneous homeostasis. Additionally, CRHR2 was predominant in rodent’s skin as compared with CRHR1, so the down-regulation of CRHR2 in lesions maybe played an important role in the pathological process. The role of CRHR2 in AD needs to be further explored.

Most importantly, we found that BSYQ treatment significantly increased the expressions of CRH and CRHR1 in the lesions of AD mice, following increased corticosterone levels and decreased inflammatory cytokine levels. However, this kind of local effect can be also regulated by central neuro-endocrine-immune systems. Previous study reported that serum CRH levels were higher and skin CRHR1 gene expressions were lower in affected samples from AD patients, as compared with healthy controls, overstimulation by the increased serum levels of CRH, leads to decreased gene expressions of skin CRH and CRHR1[36], meanwhile, activated mast cells directly[37]. In this study, we confirmed that the serum CRH level increased and local CRH in the lesion skin of AD mice down-regulated significantly. After BSYQ treatment, the levels of local CRH were significantly increased; on the contrary, the serum CRH concentrations were markedly reduced.

In skin, CRH is not only produced locally, but also can be released from sensory nerves in response to the stressor[38,39], such transport would provide a mechanism to precisely regulate CRH/POMC production, and this can explain the result of the inconsistent expression level of CRH in the AD mice skin after BSYQ treatment. CRH in skin has directly proinflammatory and indirectly immunosuppressive roles; it stimulates indirectly the production of immunosuppressive molecules, meanwhile, activates NF-κB, and stimulates expression of inflammatory cytokine production[37]. Interestingly, previous studies have found that the pro-inflammatory role of CRH was predominant in short-term of inflammatory disease; however, remote function of anti-inflammation was predominant in the chronic stage[40,41]. In this study, these AD mice were chronically stimulated with OVA, so the essential functions of elevated CRH in the lesion after BSYQ treatment were mostly related to anti-inflammatory effects.

We found that the expression of CYP11A1 mRNA were up-regulated, however, CYP11B1 were down-regulated in the lesions of AD mice, which suggested that the steroidogenesis pathway had been partially restricted. CYP11B1 was the key enzyme of corticosterone and cortisol synthesis and the situation was not significantly improved after BSYQ treatment, which indicated that therapeutic effects did not depend on this pathway. There is a close relationship among skin barrier abnormalities, immune aberrations and AD[42], however, the CYP11A1 increase cleavage of cholesterol or its precursor in the upper layer of the epidermis that would disrupt proper barrier formation[43]. Importantly, in this study the NR3C1 (GR) expressions were also down-regulated, which leading to th**e** reduced anti-inflammatory effects of glucocorticoids in the AD skins. Such changes partially contributed to endogenous corticosteroid insensitivity and skin inflammation. In conclusion, the most remarkable changes of BSYQ treatment on skin HPA axis appeared to be up-regulation of CRH and CRHR1 expression and down-regulation of POMC and CYP11A1, in addition, the down-regulated CYP11B1 and NR3C1 were slightly elevated after BSYQ treatment.

We found 16 main components in BSYQ which were Icariin, Epimedin A, B, C, Baohuoside-I and Astragaloside IV etc. Our previous studies reported that these constituents could improve HPA function[44], and displayed anti-inflammatory[45], immunomodulatory[19], and antidepressant effects[21]. This study showed that BSYQ treatment significantly alleviated abnormal symptoms and pathological changes in lesions of AD mice and presented anti-inflammatory and anti-allergic potential by systemically elevated levels of endogenous glucocorticoids and locally normalized function of skin HPA axis-like system.

In this study, central HPA axis function was only measured by basal state HPA axis tests which are generally inferior in diagnosing HPA function, while dynamic testing has the advantage of providing an assessment of stress reserve[46]. In the future, dynamic testing will be used in evaluating the central HPA axis function of AD mice. In addition, there is a close relationship between central and skin HPA axis and it is already clear that central HPA axis in communication with cytokines can regulate local steroidogenic activity and skin immune activity, however, skin as an important peripheral neuro-endocrine-immune organ is tightly networked to central regulatory systems, the effects of skin CRH/POMC on the pituitary or adrenal functions need to be further investigated.
