# Correction: BuShenYiQi Granule Inhibits Atopic Dermatitis via Improving Central and Skin Hypothalamic -Pituitary -Adrenal Axis Function

**DOI:** 10.1371/journal.pone.0126040

**Published:** 2015-05-15

**Authors:** Lingwen Kong, Jinfeng Wu, Yanhua Lin, Genfa Wang, Jia Wang, Jiaqi Liu, Meixia Chen, Xin Du, Jing Sun, Jinpei Lin, Jingcheng Dong

The second author’s name is spelling incorrectly. The correct name is: Jinfeng Wu.
